# Dementia in motor neuron disease: Reviewing the role of MRI in diagnosis

**DOI:** 10.1590/1980-57642015DN94000369

**Published:** 2015

**Authors:** Antonio José da Rocha, Renato Hoffmann Nunes, Antonio Carlos Martins Maia Jr.

**Affiliations:** 1 PhD, Division of Neuroradiology - Santa Casa de Misericórdia de São Paulo, Brazil.; 2 MD, Division of Neuroradiology - Santa Casa de Misericórdia de São Paulo, Brazil.; 3 MD, Division of Neuroradiology - Fleury Medicina e Saúde (São Paulo - Brazil).

**Keywords:** frontotemporal dementia, magnetic resonance, motor neuron disease, amyotrophic lateral sclerosis, frontotemporal lobe degeneration, demência frontotemporal, ressonância magnética, doença do neurônio motor, eslerose lateral amiotrófica, degeneração do lobo frontotemporal

## Abstract

The superimposed clinical features of motor neuron disease (MND) and frontotemporal dementia (FTD) comprise a distinct, yet not fully understood, neurological overlap syndrome whose clinicopathological basis has recently been reviewed. Here, we present a review of the clinical, pathological and genetic basis of MND-FTD and the role of MRI in its diagnosis. In doing so, we discuss current techniques that depict the involvement of the selective corticospinal tract (CST) and temporal lobe in MND-FTD.

## Abstract (pt)

As características clínicas sobreposta da doença do neurônio motor (DNM) e demência frontotemporal (DFT) compreendem um distinto ainda não totalmente compreendido, base neurológica síndrome de sobreposição clínico-patológico foi recentemente revisto. Aqui, apresentamos uma revisão das bases clínicas, patológicas e genética de DNM-DFT e o papel da ressonância magnética no diagnóstico STI. Ao fazê-lo, discutimos as técnicas atuais que retratam o envolvimento do trato corticoespinhal seletiva (TCS) e lobo temporal em DNM-DFT.

## AMYOTROPHIC LATERAL SCLEROSIS

Amyotrophic lateral sclerosis (ALS) is a fatal, late onset neurological disorder characterized by motor neuron degeneration in the primary motor cortex, brainstem and spinal cord. ALS is also known as Lou Gehrig's disease.^1,2^ The term "amyotrophic lateral sclerosis" was coined by the French neurologist Jean-Martin Charcot.^3^ Early studies of ALS, beginning in the 1880s, recognized that dementia often accompanied ALS, although this association has been largely neglected until recent years.^4^

ALS is the most common adult-onset MND and is one of the most common neurodegenerative diseases. Although familial forms of ALS have been identified, approximately 90% of cases of ALS are sporadic. Men are slightly more frequently affected than women (1.4:1).^5^ It is assumed that ALS has a relatively even distribution worldwide. It has a mean prevalence of 5.40/100,000 in Europe and 3.40/100,000 in North America.^6^ In South America, there is little information available on ALS, but it has a reported prevalence of 5.0/100,000 in Porto Alegre, Brazil.^7^ In most cases, disease onset occurs during late-adulthood (61.8 ± 3.8 years).^6^

The main neuropathological features of ALS include degeneration of the corticospinal tract (CST), extensive loss of lower motor neurons (LMN) from the anterior horns of the spinal cord and brainstem, as well as degeneration and loss of Betz cells in the primary motor cortex and reactive gliosis.^1^ Growing evidence suggests that ALS is a non-cell autonomous disease and that dysfunctional glia have an important role in the death of motor neurons. Originally, astrocytes were proposed as a central contributor to the disease,^8^ but recent data have identified equally important contributions from microglia and oligodendrocytes.^9^

A common feature of many neurodegenerative diseases, including ALS, is the formation of protein aggregates/inclusions in degenerating motor neurons. Although these pathological structures were first observed several decades ago, their presence still remains an issue of considerable debate. The exact composition of these protein structures remains largely unknown, but observed cytoplasmic inclusions containing a transactive response DNA-binding protein with a molecular weight of 43 kD (TDP-43) or fused in sarcoma (FUS), as well as their association with other ALS associated proteins, have now become hallmark pathological features of the disease.^1^ The neuronal distribution and prion-like propagation of phosphorylated TDP-43 inclusions have enabled pathologists to currently distinguish four pathological stages for ALS.^10^

In 1993, mutations in the superoxide dismutase 1 gene (*SOD1*) became the first known genetic cause of familial ALS. These mutations account for approximately 10% of all familial ALS cases.^11^ Since then, mutations in several genes have been identified as causative in ALS. Mutations in four genes (*C9orf72, SOD1, TDP-43*, and *FUS*) account for approximately 65% of familial ALS cases. Other rare genes that are causal to familial ALS include microtubule-associated protein tau (*MAPT*), progranulin (*PGRN*), valosin containing protein (*VCP*), ubiquilin2 (*UBQLN2*), and charged multivesicular protein 2B (*CHMP2B*) (Table 1).^1^

**Table 1:** A summary of genetic, clinical and brain histopathology data together with the possible target of mutations in ALS and/or FTD.^20,82^

Genes	Frequency in familial cases	Type of mutations	Brain pathology^a^	Likely pathological effect	Clinical presentation	Imaging presentation
SOD (21q22.11)	~ 20%	Mainly missense	SOD1/p62	Toxic aggregation	- Classical ALS	- Signs of UMN degeneration
FUS (16p11.2)	~5%	Mainly missense and in-frame small deletions/insertions	FUS/p62	DNA/RNA metabolism	- ALS (both juvenile- and adult-onset ALS; predominantly lower motor neuron involve­ment; rarely reported cognitive impairment)	- Signs of UMN degeneration
TARDBP (TDP43) (1p36.22)	~3%	Mainly missense and one truncating	TDP43/p62	DNA/RNA metabolism	- ALS (25% bulbar-onset; cognitive impair­ment is rarely seen)	- Signs of UMN degeneration
C9orf72 (9p21.2)	~30%	G_4_ C_2_ - repeat expansion	TDP43/p62, p62/ repeat-dipeptides, UBQLN2	Unknown (toxic RNA, toxic aggregation, low C9orf72 expression)	- ALS (bulbar ALS > 40%); - FTD (bvFTD >80%)	- Signs of UMN degeneration, - Global atrophy, may involve parieto-occipital region, thalamus and cerebellum - Less frontotemporal atrophy;
VCP (9p13.3)	Rare	Missense	TDP43/p62	Autophagy	- FTD (FTD symptoms in 30% of cases; aphasia/language deficits common); - ALS (isolated motor neuron involvement is rare, less than 2% of familial ALS cases); - Myopathy with Paget disease of bone and frontotemporal dementia	- Frontotemporal atrophy
SQSTM1 (p62) (5q35)	~3%	Missense and nonsense	TDP43/p62	Autophagy	- FTD (behavioural disorder); - ALS (limb or bulbar ALS); - Paget disease of bone (>1/3 of patients)	- Frontotemporal atrophy (may be asym­metric) - May have signs of UMN degeneration
OPTN	Rare	Missense and nonsense (haploinsufficiency)	TDP43/p62	Autophagy	- ALS; - FTD; - Glaucoma; - Paget disease of bone	–
UBQLN2 (Xp11.21)	Rare	Missense	TDP43/p62, UBQLN2, FUS, OPTN	Autophagy	- ALS, FTD (1–2% of apparent sporadic ALS and FTD; behavioural disorders precede motor symptoms); - Spastic paraplegia; - Multiple sclerosis	- Frontotemporal atrophy - May have signs of UMN degeneration
GRN (17q21.32)	~10%	Nonsense (haploinsufficiency)	TDP43/p62	Autophagy / lysosomal pathway	- FTD (Usually bvFTD (>50%); psychosis and parkinsonism are common); - Neuronal ceroid lipofuscinosis-11	- Asymmetrical frontotemporoparietal atrophy
CHMP2B (3p11.2)	Rare	C-terminal truncation of the CHMP2B	p62	Autophagy / lysosomal pathway	- FTD (early behavioural features; progressive dynamic aphasia; parkinsonism, dystonia, myoclonus, pyramidal signs later on)	- Generalized cortical atrophy at diagnosis, most marked in frontal, parietal and occipital lobes
MAPT (17q21.32)	~10%	Missense and splicing of exon 10	Abnormal tau fila­ments (tangles)	Toxic aggregation (defect in neuronal cytoskeleton)	- FTD (usually bvFTD; may be associated with other tauopathies, such as progressive supra­nuclear palsy and corticobasal degeneration)	- Relatively symmetrical orbitofrontal, medial temporal atrophy

In general, the inclusions are ubiquitin-positive and contain the ubiquitin binding protein p62. (ALS: Amyotrophic Lateral Sclerosis; FTD: Frontotemporal dementia; UMN: upper motor neuron; bvFTD= behavioural Frontotemporal dementia)

ALS is generally a pure motor disorder without any significant evidence of sensory symptoms, extraocular movement disturbances, bladder and bowel dysfunction, or cognitive impairment. The clinical diagnosis of ALS is supported by a combination of upper and LMN signs following the exclusion of "ALS mimic syndromes." ALS symptoms typically start focally, in a particular segment of the body, usually asymmetrically, before spreading to other regions over time. Bulbar onset occurs in approximately 25% of patients while respiratory onset is very rare.^1^

Upper motor neuron (UMN) signs include slow speech, brisk reflexes (brisk gag and jaw jerk, brisk limb reflexes), and Hoffman's or Babinski's signs.^2,12,13^ LMN signs include atrophy, fasciculations, and weakness. Classical ALS is diagnosed based upon the El Escorial criteria^12^ when evidence of LMN degeneration by clinical, electrophysiological or neuropathological examination is demonstrated, along with evidence of UMN degeneration by clinical examination in the absence of neuroimaging, electrophysiological or pathological evidence of a better explanation.^12,13^ Awaji criteria have recently been introduced to better define lower motor neuron degeneration, which has improved the sensitivity of early diagnostic methods for ALS. Under the Awaji criteria, needle electromyography is considered an extension of the clinical examination, but the general principles of previous criteria are maintained.^13^

## FRONTOTEMPORAL DEMENTIA

FTD is a progressive neurodegenerative condition characterized by selective involvement of the frontal and temporal lobes and is associated with changes in behavior and personality, frontal executive deficits, and language dysfunction.^14^

The first description of FTD came from Arnold Pick in 1892, who reported a patient with progressive aphasia and anterior temporal lobar atrophy.^15^Alois Alzheimer in 1911 described the pathological findings of FTD patients,^16^ specifically identifying the absence of senile plaques and the neurofibrillary tangles the had described in a disease in 1907 that bears his name. Instead, Alzheimer reported the presence of argyrophilic neural inclusions and swollen cells in FTD, later called Pick bodies and Pick cells, respectively.^16^

Once considered rare, FTD is now recognized as the second-most common early-onset dementia, affecting individuals under 65 years of age.^17^ Furthermore, there is clinical and neuropathological evidence that this condition also occurs in individuals of an advanced age.^18^ The mean age of onset of FTD is typically in the fifth to seventh decades of life.^17^

Pathologically, there is progressive degeneration of frontal and/or anterior temporal lobe neurons, which is characterized by frontotemporal lobar degeneration (FTLD).^14^ It can be divided into two major subtypes: FTLD with tau+ inclusions (FTLD-tau) and FTLD with ubiquitin+ and TDP-43+ but tau-inclusions (FTLD-TDP). Roughly, 90% of FTD syndromes show either TDP-43 proteinopathy (50%) or tauopathy. Consensus opinion currently recognizes five major pathological subtypes of FTLD (FTLD-tau, FTLD-TDP, FTLD-FUS, FTLD-UPS, and FTLD-no inclusions).^19^ The *MAPT, PGRN* and, recently, *C9orf72* genes represent the three main genetic markers associated with FTD. In addition, genetic variability in *TDP-43, CHMP2B, VCP, FUS* and transmembrane protein 10 6B (*TMEM106B*) genes contribute to <5% of cases. *MAPT, PGRN* and *C9orf72* are the major (95%) genetic markers associated with familial FTD.^20,21^

FTD is clinically characterized by different combinations of frontal lobe or frontotemporal abnormalities, including behavior changes (bvFTD) as well as gradual impairment of language skills. In this setting, primary progressive aphasia (PPA) is further subclassified into three subtypes. The most common type is a nonfluent variant, while rare logopenic and semantic dementia variants also exist.^22^

## DEMENTIA IN NOTOR NEURON DISEASE

MND is generally considered separately, and is more often free of cognitive impairment, but a growing body of evidence supports an association between MND and frontal lobe or frontotemporal dysfunction. Cognitive impairment in MND patients is correlated with pathologic and imaging abnormalities in the cerebral cortex beyond the motor regions. MND is now considered a complex multisystem neurodegenerative disease due to the discovery that areas other than the motor cortices of the brain undergo degeneration.^23^

More than 100 years after its first description, links between ALS and dementia were described as associations of ALS and dementia in Guam in specific families.^24^ The modern age of FTD and MND research began in the 1990s, when the first patients were recognized, and this marked a paradigm shift for the field.^25,26^ These reports helped to clarify that MND was associated with a specific type of dementia that is in turn associated with frontal lobe dysfunction. Conversely, the realization that MND-FTD had distinctive neuropathology began in the 1980s with the first reports of ubiquitin+ immunoreactive (UI) inclusions in the cytoplasm of motor neurons.^27,28^ In addition, evidence of UI inclusions in the extramotor cortex was shown in both pure ALS patients and ALS patients with dementia.^28^ These UI inclusions became the pathological hallmark of the combined FTD and MND syndrome.

Furthermore, in 2006 TDP-43 was identified as the major inclusion protein in this condition and is associated with UI inclusions in the vast majority of ALS patients as well as in the most common pathological subtype of FTD, now referred to as FTLD with TDP-43 pathology.^19,29^ Recognition of this mutation in *TDP-43* as being causal to ALS and FTD quickly led to screening for other RNA binding proteins.

Mutations in the *FUS* gene are now shown to account for an additional 5% of familial ALS cases and some cases of FTD.^30^ Recently, the most convincing direct molecular link between ALS and FTD has been the identification of a large, intronic hexanucleotide expansion in the previously uncharacterized *C9orf72* gene of unknown function in families with ALS, FTD, and overlapping syndrome.^31-35^ This mutation accounts for approximately 40% of familial ALS, 10% of sporadic ALS, 5% of sporadic FTD, and up to 80% of familial ALS-FTD cases, thus making it the most common cause of ALS and FTD. Many clinical MND phenotypes, including classical ALS, progressive muscular atrophy and primary lateral sclerosis, are linked to the *C9orf72* gene mutation, but generally it is characterized by bulbar-onset, cognitive impairment at a relatively early age, and accelerated disease progression.^21,33-35^ More recent insights revealing that the products of these identified genes are involved in RNA metabolism and protein homeostasis provides a further mechanistic link in the pathogenesis of this spectrum.^36^

The frequency of FTD in MND patients varies in the literature, with symptoms of FTD observed in 5-50% of ALS patients.^37,38^ Similarly, approximately 15% of FTD patients develop clinical symptoms of motor neuron dysfunction.^37^ The exact phenotype and natural history of impaired cognition in ALS remains unclear due to the heterogeneity in patient ascertainment and methods used to assess cognition. Current estimates suggest that more than half of patients with ALS have cognitive impairment. In addition to familial associations between ALS and FTD, sporadic cases of FTD in association with ALS also seem to be common,^39^ although the prevalence and etiology for this co-association remain unknown.

In some instances, FTD precedes ALS by many years; in others, ALS precedes FTD.^40^ It has been noted that a percentage of ALS patients with no previous diagnosis of FTD have early behavioural changes that precede the onset of symptoms of ALS.^41^ Several suggested risk factors for dementia in ALS include older age, male sex, lower educational level, family history of dementia, low forced vital capacity, pseudobulbar palsy, and bulbar site of onset.^38,42^

One possibility to explain the phenotypic split between the similar genetics of FTD and MND is the effect of pathological mutations on the specific function of the gene product. The genetic and pathological data, as well as mutation effect, are briefly summarized in Table 1. The strongest clinical, brain histopathology and functional overlap is observed for *VCP, OPTN, SQSTM1* and *UBQLN*2 genes, suggesting that these genes represent the core of the disease continuum.^21^ Intriguingly, mutations in three of these (*VCP, OPTN* and *SQSTM1*) cause Paget disease, in addition to ALS and FTD. These mutations are believed to cause disease by inhibiting protein degradation through autophagy and the ubiquitin-proteasome system.^20^

## SCREENING FOR MOTOR NEURON DISEASE IN FRONTOTEMPORAL DEMENTIA PATIENTS

It is critical to recognize MND associated with FTD because it greatly affects survival (8.2 years in pure FTD vs. 2.4 years in MND-FTD).^43^ Clinically, it is helpful to assess the patient for fasciculations and muscle atrophy, which are non-specific features but if present, might indicate a need for further testing. Signs of muscle weakness, spasticity, or bulbar involvement should be extensively explored. Ultimately, in suspected cases, electroneuromyography should be performed because it is considered the most sensitive measure of LMN involvement. Additionally, this test can identify early neuron loss before clinical weakness is noted. The tongue muscle should also be studied because ALS can start in any one of the four limbs or in the bulbar region. ^44^ Alternately, fasciculations may be demonstrated by muscle ultrasound which is considered a feasible, reliable and well tolerated non-invasive technique for defining LMN involvement in FTD patients.^45,46^. It is important to take care to exclude ALS mimetic syndromes if any abnormalities, such as spinal disease or neuropathy, are found, because a diagnosis of MND in FTD is otherwise fatal.^44^

## SCREENING FOR FRONTOTEMPORAL DEMENTIA IN MOTOR NEURON DISEASE PATIENTS

The prevalence of FTD in MND ranges from 22% to 48%.^41^ This variability depends, in part, on how FTD is classified and whether more subtle signs of FTD are included. If strict Neary criteria are used, then 22% is a more accurate figure.^38^ ALS patients who are clearly not normal but have cognitive or behavioural disturbances that do not match the strict Neary criteria should be classified based on the Strong et al. classification (Table 2).^47^

**Table 2:** Defining cognitive and behavioural subtypes in ALS.^83^

ALS-FTD	**ALS-bvFTD**	ALS patient meeting either the Neary criteria or Hodge's criteria for FTD
	**ALS-PNFA**	ALS patient meeting Neary criteria for PNFA
	**ALS-SD**	ALS patient meeting Neary criteria for SD
Other forms	**ALSbi**	ALS patient meeting at least two non-overlapping supportive diagnostic features from either the Neary criteria or Hodge's criteria for FTD
	**ALSci**	Evidence of cognitive impairment at or below the 5th percentile on at least two different tests of cognition that are sensitive to executive functioning
	**FTD-MND like**	A neuropathological diagnosis with a primary frontotemporal lobar degeneration diagnosis with evidence of MND-type degeneration but insufficient to be classified as ALS
	**ALS dementia**	ALS with dementia, not typical of FTD (ALS-Alzheimer, ALS-vascular dementia)
	**ALS–parkinsonian dementia complex**	ALS concurrent with dementia and/or parkinsonianism occurring in hyperendemic foci of the Western Pacific

ALS: Amyotrophic Lateral Sclerosis; FTD: Frontotemporal Dementia; ALS-bvFTD: Amyotrophic Lateral Sclerosis and behavioural Frontotemporal Dementia; ALS-PNFA= Amyotrophic Lateral Sclerosis and Primary Non-fluent Aphasia; ALS-SD: Amyotrophic Lateral Sclerosis and Semantic Dementia; ALSbi: Amyotrophic Lateral Sclerosis with behavioural impairment; ALSci: Amyotrophic Lateral Sclerosis with cognitive impairment; MND: Motor Neuron Disease.

A myriad of different cognitive screening exams have been developed centering on the need to develop shorter measures, given that a full neuropsychological battery is hard for patients to tolerate, particularly if they have advanced disease. Each of these tests are suited to different situations because they have benefits and drawbacks in their utility.^44^ It is also important to consider alternative explanations when cognitive abnormalities are observed. For example, while depression is unusual in ALS, it could certainly be a cause of apathy and other underlying psychiatric disorders that mimic FTD. Pseudobulbar syndrome is rarely confused with FTD, but it can affect some of the behavioural measures and even interfere with testing when severe because patients with this problem have extreme difficulty controlling their emotions. Pseudobulbar syndrome also tends to be more common in MND-FTD than in MND alone because it is more commonly found in bulbar onset patients, who are more likely to have FTD.^36,38,44^

## NEUROIMAGING FINDINGS

While some doubts remain over whether MND-FTD is nosologically distinct or part of a spectrum of diseases ranging from classic MND to FTD at the end of its presentation, neuroimaging studies have provided significant insight into the biological basis of the FTLD syndromes in MND patients. Including both morphologic and functional neuroimaging, MRI has largely validated the hypothesis that MND is a multi-system disorder with brain involvement well outside of the motor system.

There are many historical reports of FTD that have been superimposed onto MND, although until recently, the clinicopathological entity of this syndrome had been controversial from neuropsychological and neuropathological perspectives. Neuroimaging features have been reported, and in addition to MND, degeneration in the frontal and temporal lobes is consistently observed as a pathological feature.^48^

It is now commonly thought that MND and FTD represent a continuum,^41^ and even in ALS patients, who are cognitively normal, MRI shows the presence of abnormalities in the frontal and temporal lobes. Although the atrophy is not as severe as that seen in ALS patients with cognitive abnormalities, the anatomical areas of involvement are clearly identical. A recent paper describing a family with ALS and FTD similarly shows a degree of involvement that depends upon the severity of FTD in the MND cases.^49^

In this context, Mori et al.^50^ compared structural MRI findings of ALS patients with dementia (ALSD) and without dementia to identify a pattern that would distinguish both. Patients with ALSD showed bilateral frontotemporal atrophy mostly with temporal lobe dominance. In addition, in the ALSD group, T2-weighted imaging (T2WI) disclosed hyperintensity in the subcortical white matter on the medial side of the anterior temporal lobes, whereas in the group without dementia, no patients exhibited this imaging finding. The authors, however, did not distinguish groups of patients based on the genetic profile, which might have interfered with the groups and the neuroimaging findings analysis.

The relentless pursuit of structural biomarkers of disease has been fruitful. Volumetric studies have shown a reasonable, although imperfect, correlation between the presence of dementia and the occurrence of atrophy in the frontal and anterior temporal lobes, which is then followed by atrophy of the anterior cingulate gyrus.^51-53^ Lillo et al.^54^ investigated grey and white matter changes across the ALS-FTD continuum and observed that all clinical syndromes showed grey matter changes in motor cortical and anterior cingulate brain regions. Although clinical syndromes display considerable atrophy overlap, there are also atrophy patterns specific to each subtype of the continuum. More substantial prefrontal and temporal cortex atrophy was indicative of bvFTD when compared to ALS and ALS-FTD, while ALS-FTD showed substantially more anterior cingulate and anterior temporal lobe grey matter atrophy when compared to ALS. Patients with ALS-FTD due to *C9ORF72* mutation demonstrate symmetric frontal and temporal lobe, insular, and posterior cortical atrophy, although temporal involvement may be less than that seen in other mutations. Diffuse cortical atrophy, that includes anterior as well as posterior structures and subcortical involvement, may therefore represent unique features of this mutation.^55^

Avants et al.^56^ used high-resolution diffeomorphic image normalization and serial MRI to provide the first assessment of longitudinal cortical atrophy in patients with ALS-FTD relative to controls. Significant abnormalities were documented in the premotor cortex, primary motor cortex, and parietal lobe bilaterally in Brodmann areas (BA) 4, 6, and 7. The average annual cortical atrophy over significant voxels in ALS-FTD on the right and left was 8.5% and 7.6% in BA4, respectively; 8.1% and 5.9% in BA6; and 3.6% and 2.2% in BA7. For all cortices in ALS-FTD patients, the atrophy rate was 1.0% per year whereas in elderly controls, the atrophy rate was 0.25% per year. The local atrophy rate did not correlate with overall brain atrophy while age and overall brain atrophy rates also did not correlate.

To outline the difficulties of implementing sophisticated volumetric techniques in everyday clinical practice due to time and cost restraints, Ambikairajah et al.^51^ proposed a simple coronal MRI atrophy rating scale. The authors argued that ALS, ALS-FTD, and bvFTD patients can be distinguished by analysing four cortical grey matter regions: the motor cortex, the anterior cingulate gyrus, the anterior temporal lobe, and the orbitofrontal cortex. The authors demonstrated that bvFTD patients showed the highest levels of atrophy across all regions, while ALS patients had the lowest atrophy scores. ALS-FTD patients have higher atrophy ratings compared with ALS patients for the motor cortex, anterior cingulate gyrus and anterior temporal lobe, with a statistical tendency for the orbitofrontal cortex. ALS-FTD patients did not differ significantly to bvFTD patients for any of the brain regions.

Over the last decade, conventional MRI was considered to have low specificity for the diagnosis of MND; however, non-conventional MRI, including magnetization transfer imaging, have shown dynamic utility in this setting.^57^ More recently, we have demonstrated the use of T1-weighted spin-echo magnetization transfer contrast (T1 MTC) sequences to detect selective CST involvement when UMN phenotype is documented in ALS patients, particularly in early disease.^58,59^ Moreover, although initially considered to be a marker of the UMN components of ALS, particularly in advanced disease, a thin line of cortical low signal intensity ("motor dark line" or "hypointense rim") of the precentral gyrus on T2WI or Fluid-attenuated inversion recovery (FLAIR) images is neither sensitive nor specific for the pathology of UMN degeneration in ALS and can be found in healthy individuals as well as in those with other degenerative diseases.^60^ Both of these imaging findings are less prevalent in ALS-FTD patients.^61^ When present, although inconsistent, the MRI characterization of the CST and motor cortex degeneration is often relatively mild, thereby leading to fewer represented imaging findings in these regions.

In a recent study of the anatomical and radiological correlation in relevant diseases, Mori et al.^50^ demonstrated that signal-intensity changes on T2WI were observed more frequently in the pre-central white matter than in the posterior limb of the internal capsule. However, it is notable that the authors used fast spin-echo T2WI, whose sensitivity for disclosing CST impairment is less than ideal. Conventional T2WI MR acquisitions have low sensitivity (about ≤ 40%) and limited specificity (about ≤ 70%)^62^ in demonstrating areas of abnormal signal intensity in the CST, and these abnormalities have proven inconsistent and unreliable because they were observed frequently in normal patients and invariably did not correlate with clinical scores.

Conversely, magnetization transfer imaging (MTI) is based upon the exchange of magnetization between spins in the two different pools of protons: bound immobile protons associated with macromolecules (such as myelin) and free mobile protons associated with free water.^63^ CST hyperintensity in ALS patients, particularly in the supratentorial compartment, on T1 MTC was reported with great sensitivity (80%) and specificity (100%).^58,63^ This sequence has fast and simple acquisition, and is particularly useful in early MND with UMN signs to demonstrate abnormally selective hyperintensity throughout the CST, crossing the corpus callosum, assuming a typical 'W-like appearance'.^59,63-65^

**Figure 1:**
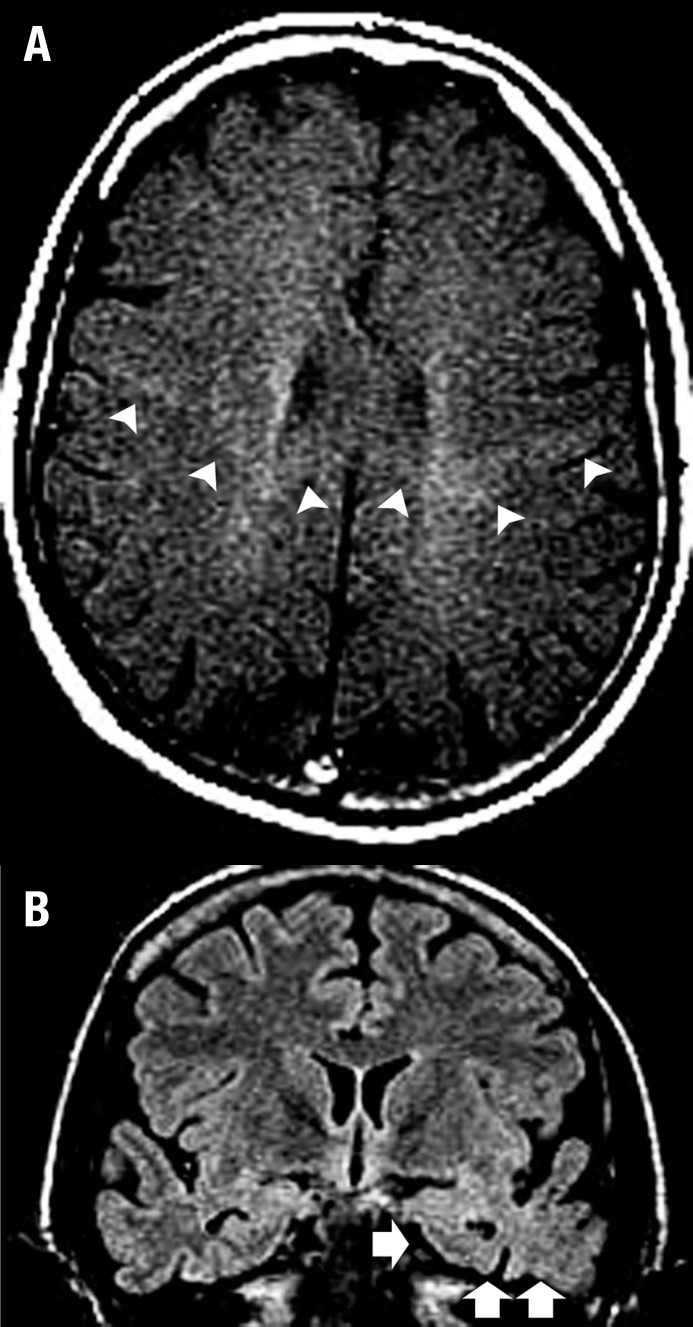
A man of 42 years of age presented with UMN + LMN signs associated with dementia and language-speech abnormalities (his mother had died at 67 years old with a diagnosis of ALS-FTD). [A] Axial T1 MTC image showed an abnormally selective hyperintensity throughout the corticospinal tracts, predominantly on the left side, assuming a typical 'W-like appearance' crossing the corpus callosum (arrowheads). [B] Coronal FLAIR image depicted a marked atrophy in the left temporal pole with blurring of the grey/white matter differentiation (arrows). Note the abnormal hyperintensity in the left amygdala and hippocampus.

**Figure 2:**
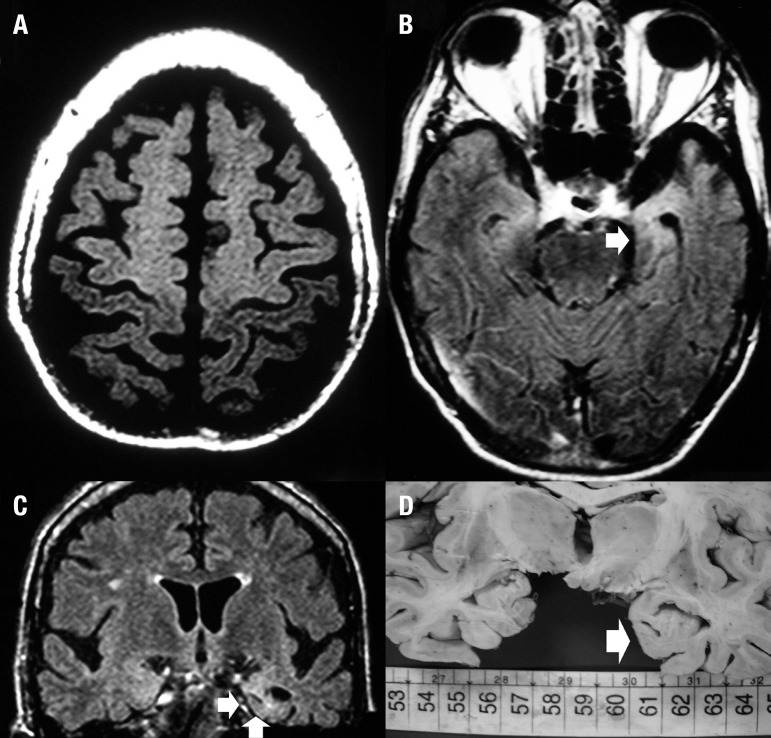
A man of 61 years of age presented with a history of progressive non-fluent aphasia in the four months prior to admission. This was followed by an asymmetrical flaccid tetraparesis, hyperreflexia in all four limbs, which was associated with a bilateral Hoffmann sign, and fasciculation. [A] Axial T1 MTC image showed an abnormal hyperintensity involving the cortical and subcortical frontal areas including pre-central gyri and the remaining subcortical regions of the frontal lobes. [B-C] Axial and coronal FLAIR images depicted left temporal lobe atrophy with blurring of the grey/white matter differentiation (arrows). [D] Brain macroscopy confirmed atrophy in the left temporal lobe (arrow). Left hippocampus cortex showed neuronal loss as well as cytoplasmic, nuclear and extracellular UI inclusions, in addition to neuronal loss and astrogliosis (not shown).

Complementary to clinical and neuropsychological evaluations, the association of asymmetrical cortical atrophy and CST composition provides key information for the imaging diagnosis of ALS-FTD (Figure 1). Preliminary studies in pathologically proven cases suggested that distinct patterns of tissue loss could assist in predicting pathological subtype *in vivo*.^53^ We have reported the combined involvement of both dominant frontal and temporal lobes, as well as bilateral CST involvement, as determined by MRI *in vivo* using T1 MTC sequence and T2/FLAIR images. Our pathological findings also predominated in the same sites depicted by these MRI sequences.^66^ While T1 MTC is able to demonstrate motor and extra-motor involvement in both CST and frontal lobes and is positively coincident to brain injury, as reflected in the UI distribution, T2/FLAIR images are useful for demonstrating subcortical gliosis, as confirmed by histopathological analysis (Figure 2).

Structural MRI studies have shown that bvFTD typically presents asymmetrically with a combination of frontal and anterior temporal cortical atrophy. Patterns of brain atrophy are likely to be associated with the different pathological substrates of bvFTD.^67^ On structural MRI, each PPA variant is associated with a specific pattern of focal atrophy: left frontoinsular and peri-sylvian atrophy in the non-fluent variant, asymmetric atrophy of the anterior temporal and ventromedial frontal lobe in the semantic variant, and left temporoparietal atrophy in logopenic patients.^67,68^ Conversely, subjects who present with prosopagnosia also present with predominantly right temporal lobe atrophy. This pattern of atrophy can also be observed in patients who present with predominantly behavioural features and in those who have prominent geographic disorientation.^69,70^

In addition to the morphological alterations outlined, functional studies have encouraged the view of a continuum between ALS and FTD. In a prospective study of cognition in ALS across two time points separated by six months, Strong et al.^71^ observed a significant loss of neurons (as indicated by a reduction in the NAA/Cr ratio) in the anterior cingulate gyrus that preceded a significant loss of motor neurons in the precentral gyrus (motor cortex). The loss of anterior cingulate gyrus neurons was correlated with impairments in verbal praxis, a feature consistent with previous clinical and functional neuroimaging studies. In addition, Abrahams et al.^72^ observed a significant impairment in functional MRI (fMRI) activation in the middle and inferior frontal gyri and anterior cingulate gyrus with tasks of letter fluency. These findings were interpreted as suggestive of cerebral abnormalities in ALS in networks of regions involved in language and executive function.

Frontotemporal hypoperfusion (SPECT) in anterior and inferior regions to the primary cortex and hypometabolism (PET), mainly in the thalamo-frontal association pathways in some non-demented ALS patients, as well as dysfunction of the dorsolateral prefrontal cortex in ALS patients with associated cognitive impairment (i.e., deficits in letter fluency, executive and memory dysfunction) have been described in cognitively unimpaired patients with ALS. This indicates that an extension of cerebral involvement accompanies cognitive impairment in ALS, with a pattern shared by FTD; however, these symptoms do not completely overlap.^73-76^

Diffusion tensor imaging (DTI) provides quantitative information about the magnitude and directionality of water diffusion in 3D space and has been used to assess patients with isolated forms of ALS (without dementia) and FTD (without motor symptoms).^77^ Decreased fractional anisotropy (FA) in ALS patients was found to correlate with several clinical aspects of the disease.^78-80^ Conversely, in bvFTD, DTI abnormalities involved preferentially white matter tracts located in the frontal lobes and those passing through the temporal lobes. Nevertheless, diffusivity changes were also identified in more posterior white matter regions.^67^

Using voxel-based morphometry (VBM) and DTI analysis of brain MRI to examine grey and white matter differences and commonalities across the continuum, Lillo et al.^54^ demonstrated that, in comparison to controls, bvFTD showed substantial degeneration in the forceps minor, anterior corpus callosum, anterior inferior longitudinal fasciculus and CST. Similarly, ALS-FTD patients showed white matter degeneration in the same tracts as bvFTD but to a lesser degree in the forceps minor and anterior corpus callosum. The authors also demonstrated that a more anterior portion of the inferior longitudinal fasciculus was affected, and the CST more substantially degenerated. As expected, ALS patients demonstrated more substantial changes in the CST compared to controls, while only mild abnormalities were observed in the forceps minor, anterior corpus callosum and the inferior longitudinal fasciculus.

Advanced MRI techniques hold the promise of capturing UMN loss as well as extramotor brain abnormalities in MND and as such deliver biomarkers relevant to diagnosis.^63^ Nevertheless, a correlation between imaging parameters and clinical metrics has thus far been inconsistent across studies.^80,81^

We argue that T1 MTC should be routinely included in the workup of patients with weakness and pyramidal signs as a sensitive and accurate imaging acquisition approach useful for depicting CST involvement in ALS suspected patients.^58,63,64^ Structural MR sequences, including FLAIR and 3D acquisitions, are also recommended when extra-motor involvement is suspected, considering the MND/FTD spectrum.^66,68^ Further investigations using structural and nonconventional techniques are recommended to identify MR features in MND-FTD patients and to correlate *in vivo* abnormalities with neuropathological diagnostic criteria. Future genetic, clinicopathological and biochemical results remain necessary for fuller comprehension of the MND-FTD spectrum, while determining the imaging correlation amongst clinical, imaging, and histopathologic features in this overlapping syndrome is highly desirable.

## CONCLUSIONS

Neuroimaging studies have provided consistent evidence for a more diffuse metabolic derangement in MND that extends well beyond traditional 'motor neuron specific' domains. These findings not only support the concept of MND as syndromic but have also confirmed the widespread involvement of the disease process.

Despite each MND-FTLD variant being associated with characteristic behavioural and/or linguistic features, the fact that they harbour different underlying pathological processes renders the diagnostic work-up of these patients a highly challenging task. Nevertheless, when associated with motor neuron damage visible on MTI, the detection of distinct patterns of atrophy and/or subcortical gliosis on combined structural and nonconventional MRI, mainly using T2/FLAIR, 3D-T1WI, DTI and VBM, in addition to functional abnormalities on SPECT and PET scans, have been shown to contribute to support a correct diagnosis of MND-FTD.
